# Regulation of Autophagy by Protein Kinase C-ε in Breast Cancer Cells

**DOI:** 10.3390/ijms21124247

**Published:** 2020-06-15

**Authors:** Alakananda Basu

**Affiliations:** Department of Microbiology, Immunology and Genetics, University of North Texas Health Science Center, Fort Worth, TX 76107, USA; Alakananda.basu@unthsc.edu; Tel.: +1-817-735-2487

**Keywords:** PKCε, autophagy, apoptosis, breast cancer, mTORC1, mTORC2, Raptor, Rictor

## Abstract

Protein kinase C-ε (PKCε), an anti-apoptotic protein, plays critical roles in breast cancer development and progression. Although autophagy is an important survival mechanism, it is not known if PKCε regulates autophagy in breast cancer cells. We have shown that silencing of PKCε by siRNA inhibited basal and starvation-induced autophagy in T47D breast cancer cells as determined by the decrease in LC3-II, increase in p62, and decrease in autophagy puncta both in the presence and absence of bafilomycin A1. The mechanistic target of rapamycin (mTOR) associates with Raptor or Rictor to form complex-1 (mTORC1) or complex-2 (mTORC2), respectively. Knockdown of PKCε attenuated an increase in autophagy caused by the depletion of Raptor and Rictor. Overexpression of PKCε in MCF-7 cells caused activation of mTORC1 and an increase in LC3-I, LC3-II, and p62. The mTORC1 inhibitor rapamycin abolished the increase in LC3-I and p62. Knockdown of mTOR and Rictor or starvation enhanced autophagy in PKCε overexpressing cells. While overexpression of PKCε in MCF-7 cells inhibited apoptosis, it induced autophagy in response to tumor necrosis factor-α. However, inhibition of autophagy by Atg5 knockdown restored apoptosis in PKCε-overexpressing cells. Thus, PKCε promotes breast cancer cell survival not only by inhibiting apoptosis but also by inducing autophagy.

## 1. Introduction

Protein kinase C (PKC) is a family of serine/threonine protein kinases that play critical roles in signal transduction and cell regulation [1]. Based on the structural variations and biochemical properties, the PKC family can be categorized into three groups: Conventional (α, βI, βII, and γ), novel (δ, ε, η, and θ), and atypical (λ/ι and ξ) [2]. PKCε, a member of the novel PKC family, was identified as a transforming oncogene [3]. It is overexpressed in several cancers, including breast cancer, and has been associated with cancer development and progression [4,5,6]. PKCε also promotes cell survival by functioning as an anti-apoptotic kinase [3,7,8,9,10]. Recent studies implicate PKC isozymes, including PKCε, in autophagy [11,12].

Autophagy is a process of cellular cannibalism by which cells recycle their own components to survive under stressful or nutrient-deprived conditions [13]. During autophagy (or macroautophagy), cytoplasmic constituents or damaged organelles are sequestered in double membrane vesicles called autophagosomes, which subsequently fuse with lysosomes, where enclosed materials are degraded and recycled [14]. While basal autophagy functions as a clearance mechanism to remove damaged organelles and long-lived proteins, it is activated during cellular stress [15]. Although autophagy is considered a cell survival mechanism, it can also contribute to cell death depending on the nature and duration of the stress [16].

The mechanistic target of rapamycin (mTOR) is considered the master regulator of autophagy [14]. mTOR forms two complexes: mTOR complex 1 (mTORC1) and mTOR complex 2 (mTORC2) [17]. The most distinguishing feature of the two complexes is the binding of the adaptor protein Raptor with mTORC1 and Rictor with mTORC2 [17]. It is believed that during nutrient deprivation, inhibition of mTORC1 triggers autophagy [14,18]. While mTORC1 functions downstream of the Akt signaling pathway, mTORC2 functions upstream of Akt and PKCε [17,19,20]. 

We previously showed that overexpression of PKCε inhibited tumor necrosis factor-α (TNF)-induced apoptosis in breast cancer MCF-7 cells [7,8,21]. Although autophagy plays an important role in breast cancer [22], it is not known if PKCε promotes breast cancer cell survival via autophagy. In the present study, we investigated if PKCε regulates autophagy in breast cancer cells and if induction of autophagy is associated with PKCε-mediated cell survival.

## 2. Results

### 2.1. The Effect of PKCε on Basal Autophagy

**Figure 1:**
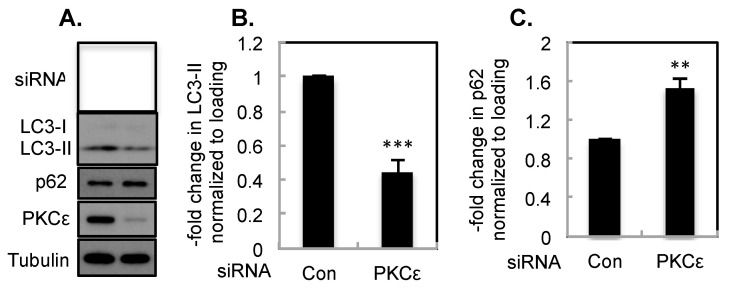
PKCε knockdown inhibits basal autophagy. T47D cells were transfected with control non-targeting or PKCε siRNA. (**A**) Western blot analysis was performed with indicated antibodies. Tubulin was used to control for loading differences. Bar graph represents mean ± S.E of LC3-II (**B**) and p62 (**C**) from several independent experiments. ***, *p* ≤ 0.0005 (*n* = 10); **, *p* ≤ 0.005 (*n* = 7).

During autophagy, MAP1LC3/LC3, which exists in the cytoplasm as LC3-I, is conjugated to phosphatidylethanolamine to form LC3-II and is recruited to the autophagosomal membrane [23,24]. LC3-II migrates faster on SDS-PAGE (sodium dodecyl sulfate-polyacrylamide gel electrophoresis) compared to LC-I [23,24]. In addition, sequestosome 1 (SQSTM1)/p62 binds to LC3 as well as ubiquitinated proteins to target them for degradation but itself becomes degraded in the process [23,24]. Thus, an increase in LC3-II and a decrease in p62 serve as markers for autophagy. Since T47D cells contain high levels of PKCε and also exhibit high basal levels of autophagy, we examined if silencing of PKCε by siRNA affects LC3-II and p62 levels in T47D cells. As shown in Figure 1A, knockdown of PKCε caused a decrease in LC3-II and an increase in p62. Based on several independent experiments, the decrease in LC3-II (Figure 1B) and increase in p62 (Figure 1C) were statistically significant. These results suggest that depletion of PKCε inhibits basal autophagy.

**Figure 2:**
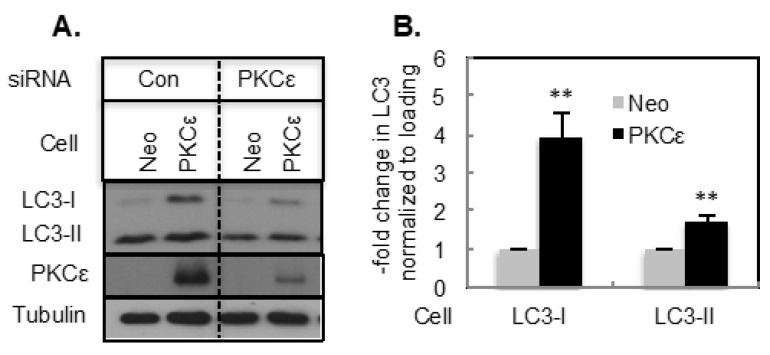
Overexpression of PKCε increased LC3-I and LC3-II. (**A**) Western blot analysis was performed in MCF-7 cells stably transfected with a vector containing neomycin without (Neo) or with a PKCε construct (PKCε) and transfected with control or PKCε siRNA. (**B**) Bar graph represents mean ± S.E of LC3-I and LC3-II levels **, *p* ≤ 0.005 (*n* = 9). The comparison was made between MCF-7/Neo and MCF-7/PKCε cells.

We then examined if overexpression of PKCε affects basal autophagy. We generated MCF-7 cells stably expressing PKCε [21] and showed that PKCε overexpression inhibits apoptosis [7,8,21]. We therefore examined if autophagy is also affected in these cells. Overexpression of PKCε in MCF-7 cells (MCF-7/PKCε) enhanced both LC3-I and LC-II levels compared to cells transfected with the vector alone (MCF-7/Neo) (Figure 2A,B). The increase in LC3-I was much more pronounced compared to the increase in LC3-II (Figure 2B). Knockdown of PKCε decreased both LC3-I and LC3-II in MCF-7/Neo and MCF-7/PKCε cells (Figure 2A).

### 2.2. The Effect of PKCε on Starvation-Induced Autophagy

**Figure 3:**
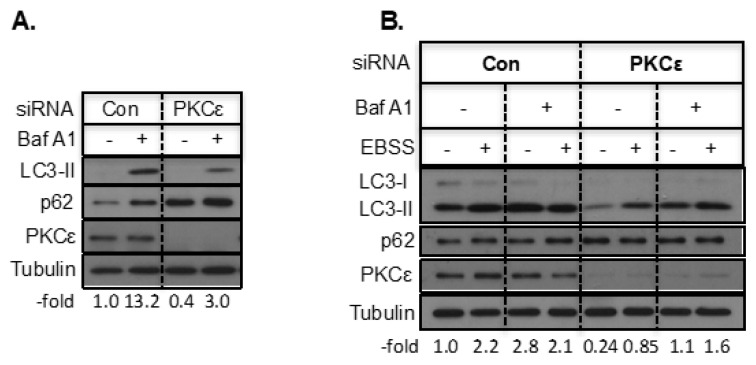
Knockdown of PKCε inhibited starvation-induced atuophagy. T47D cells transfected with control or PKCε siRNA were treated with Baf A1 (**A**) or EBSS in the presence or absence of Baf A1 (**B**). The fold change in LC3-II with respect to control and normalized with loading is indicated below the Figures.

A decrease in LC3-II could be due to not only a decrease in autophagosome formation but also an increased degradation of LC3-II following autophagosome–lysosome fusion. We therefore monitored LC3-II levels in the presence and absence of bafilomycin A1 (Baf A1), which inhibits both the fusion of autophagosomes with lysosomes and lysosomal acidification. As shown in Figure 3A, treatment of T47D cells with Baf A1 caused an increase in LC3-II as well as p62 but knockdown of PKCε attenuated the increase in LC3-II by Baf A1 and further increased p62. We then examined if knockdown of PKCε inhibits starvation-induced autophagy. Starvation of T47D cells in Earle’s balanced salt solution (EBSS) enhanced LC3-II and knockdown of PKCε decreased the starvation-induced increase in LC3-II both in the presence and absence of Baf A1 (Figure 3B). However, low concentrations of Baf A1 (10 nM) used in this study may not completely block LC3-II turnover when control siRNA-transfected T47D cells were starved in EBSS (Figure 3B). These results suggest that PKCε knockdown inhibits starvation-induced autophagy in T47D cells. 

**Figure 4:**
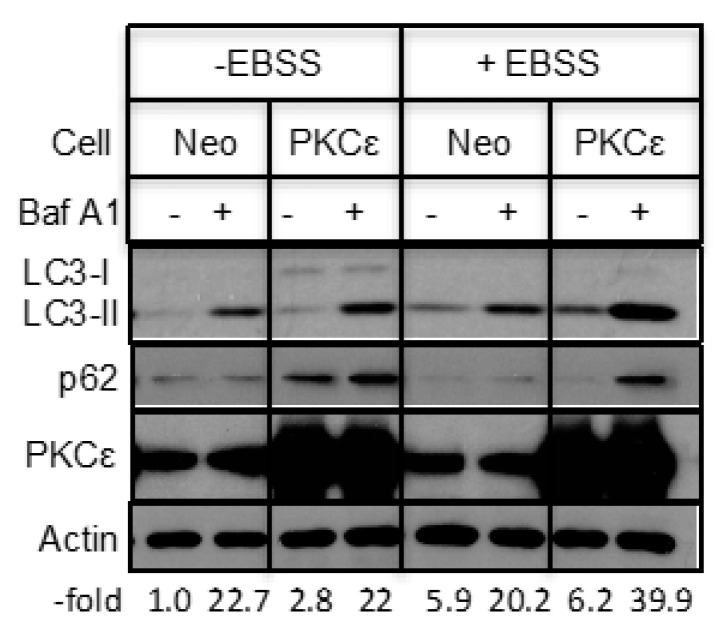
PKCε overexpression enhanced starvation-induced autophagy. MCF-7/Neo and MCF-7/PKCε cells were starved in EBSS in the presence or absence of Baf A1 and Western blot analyses were performed with indicated antibodies.

We also examined if overexpression of PKCε affects starvation-induced autophagy. Since an increase in LC3-II in PKCε-overexpressing cells could be due to a decrease in the fusion of autophagosomes with lysosomes or inefficient turnover of the cargo, we performed the experiment in the absence and presence of Baf A1. Figure 4 shows that treatment with Baf A1 alone had little effect on LC3-II in PKCε-overexpressing cells but starvation in EBSS caused an increase in LC3-II in Baf A1-treated MCF-7/PKCε cells compared to MCF-7/Neo cells.

**Figure 5:**
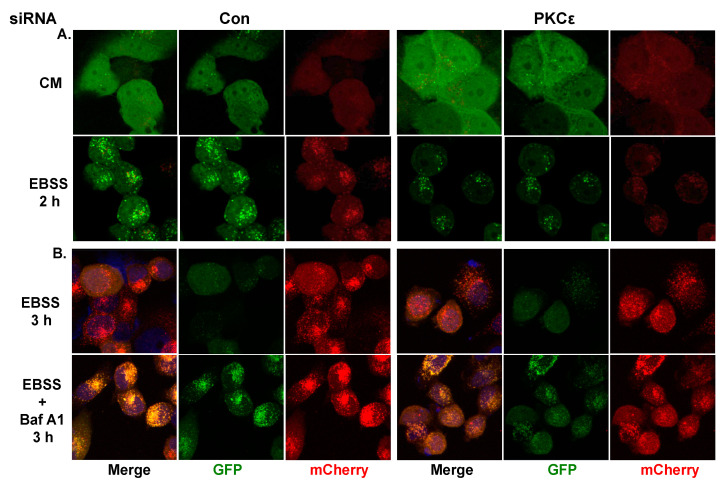
PKCε knockdown decreased starvation-induced autophagic flux. T47D cells expressing mCherry-GFP-LC3 were transfected with control or PKCε siRNA and then grown in either complete media or in EBSS for 2 h (**A**). (**B**) Cells were incubated in EBSS for 3 h in the presence or absence of 100 nM Baf A1.

An alternate and more definitive way to monitor autophagy is to visualize the appearance of autophagic puncta by fluorescence microscopy [23,24]. When LC3 is incorporated into autophagosomes, the diffused pattern of LC3-I in the cytosol is changed to a distinct punctate structure. We stably expressed a tandem mCherry-GFP-LC3 construct in T47D cells (T47D-LC3) to visualize LC3 in the autophagosomes as well as in lysosomes. While GFP green fluorescence is pH sensitive, mCherry red fluorescence is stable at low pH [24]. Therefore, autophagosomes are marked by the presence of both green and red fluorescence whereas only red fluorescence could be detected in late endosomes or lysosomes. We transfected T47D-LC3 cells with control non-targeting or PKCε siRNA. Prior to processing cells for confocal microscopy, cells were grown in either complete media or starved by culturing them in EBSS. As shown in Figure 5A, autophagy puncta could not be detected in cells transfected with control or PKCε siRNA when grown in complete media. Both green and red puncta increased substantially in control siRNA-transfected T47D cells when grown in the presence of EBSS but attenuated in cells transfected with PKCε siRNA. When cells were starved in EBSS for 3 h, the autophagy puncta were primarily red, suggesting their localization in the late endosomes/lysosomes (Figure 5B). However, when Baf A1 was included with EBSS, we could detect both red and green puncta, suggesting their localization in autophagosomes. Knockdown of PKCε resulted in a substantial decrease in both red and green puncta. These results suggest that PKCε positively regulates EBSS-induced autophagy.

### 2.3. The Effect of PKCε on Autophagy Mediated by the mTOR Signaling

**Figure 6:**
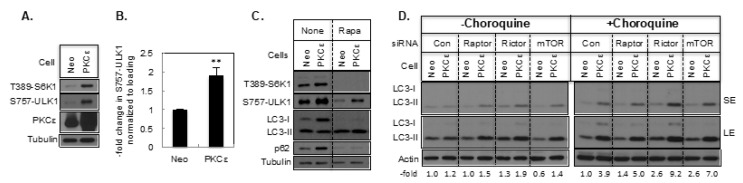
The effect of PKCε overexpression and mTOR signaling on autophagy. (**A**) Western blot analysis was performed with MCF-7/Neo and MCF-7/PKCε cells. (**B**) Bar graph represents mean ± S.E. **, *p* ≤ 0.005 (*n* = 10). (**C**) Effect of Rapamycin on LC3-I, LC3-II and p62. Cells were treated with or without 100 nM Rapamycin and Western blot analysis was performed. (**D**) MCF-7/Neo and MCF-7/PKCε cells were transfected with control non-targeting, Raptor, Rictor or mTOR siRNA and then treated with or without 10 µM chloroquine for 2 h. Western blot analyses were performed with indicated antibodies. Actin was used to control for loading differences. SE, short exposure; LE-long exposure. The fold change in LC3-II with respect to control and normalized with loading is indicated below the Figures.

Since mTORC1 is the master regulator of autophagy, we examined if PKCε regulates autophagy via mTORC1. Overexpression of PKCε increased phosphorylation of its substrate S6K1 at the Thr389 site (Figure 6A). However, this antibody also recognizes PKCε phosphorylated at the hydrophobic motif site. In nutrient-rich conditions, when mTORC1 is active, it phosphorylates ULK1 at Ser757 to inhibit autophagy [25,26]. We therefore examined if PKCε regulates ULK1 phosphorylation. Overexpression of PKCε in MCF-7 cells was associated with an increase in S757-ULK1 phosphorylation (Figure 6A,B). Since inhibition of mTORC1 rather than activation of mTORC1 is associated with an increase in autophagy, we examined the effect of the mTORC1 inhibitor rapamycin on the PKCε-mediated increase in LC3-I and LC3-II. Rapamycin inhibited phosphorylation of mTORC1 substrates as well as the abundance of LC3-I and p62 (Figure 6C), suggesting that activation of mTORC1 by PKCε may be responsible for the increase in LC3-I and p62. Since rapamycin is a pharmacological inhibitor, we examined how silencing of mTOR as well as the mTORC1 and mTORC2 components Raptor and Rictor affect LC3-II levels in MCF-7/Neo and MCF-7/PKCε cells.

We performed the experiment both in the absence and presence of chloroquine, a lysosomotropic agent. Knockdown of mTOR, Raptor, and Rictor had little effect on the increase in LC3-II in MCF-7/Neo cells but enhanced LC3-II in MCF-7/PKCε cells when chloroquine was also present (Figure 6D). Moreover, Rictor knockdown was more effective in enhancing LC3-II compared to Raptor or mTOR knockdown.

**Figure 7:**
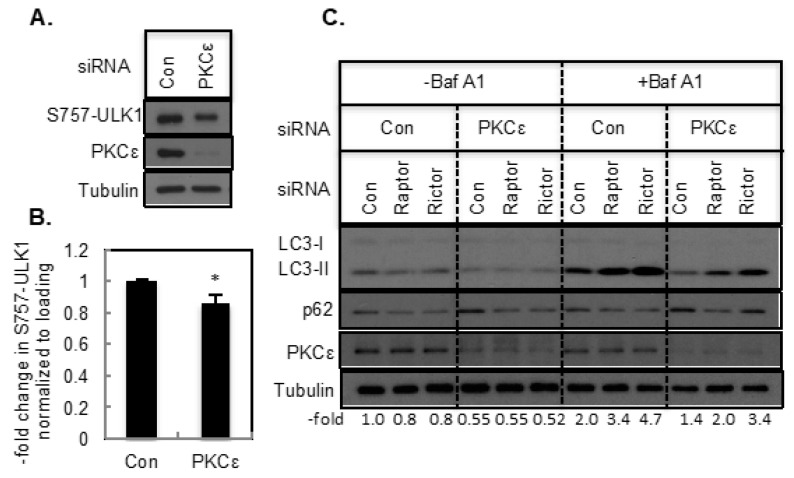
The effect of PKCε, Raptor and Rictor knockdown on autophagy. T47D cells were transfected with control or PKCε siRNA. (**A**) or double transfected with Raptor and Rictor siRNA (**B**) Bar graph represents mean ± S.E. *, *p* ≤ 0.05 (*n* = 11). (**C**) Western blot analyses were performed with indicated antibodies.

We then examined how knockdown of PKCε affects mTORC1 signaling. PKCε knockdown appears to decrease S757-ULK1 phosphorylation slightly (Figure 7A,B). While both Raptor and Rictor knockdown enhanced LC3-II in control siRNA-transfected cells in the presence of Baf A1, PKCε knockdown attenuated this increase (Figure 7C). Moreover, Rictor knockdown was more effective in enhancing LC3-II compared to Raptor knockdown (Figure 7C). These results suggest that knockdown of PKCε inhibits autophagy induced by the depletion of Raptor and Rictor. 

### 2.4. The Effect of PKCε-Mediated Autophagy on Apoptosis

**Figure 8:**
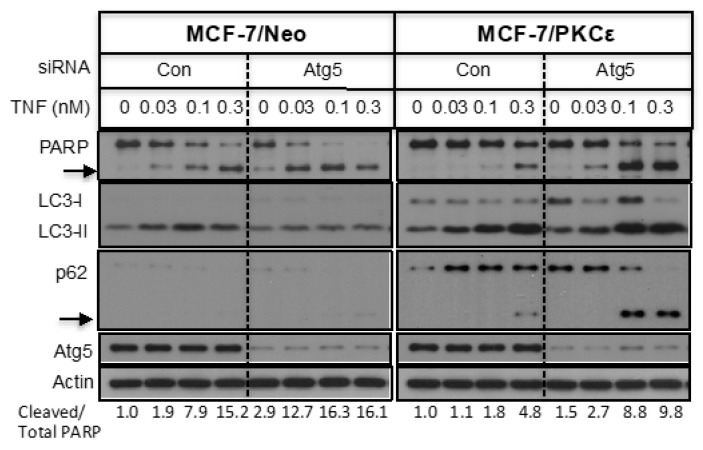
PKCε promotes cell survival by inhibiting apoptosis and inducing autophagy. Cells were transfected with control or Atg5 siRNA and then treated with different concentrations of TNF. Western blot analyses were performed with indicated antibodies. The arrow indicates cleaved fragments.

We have previously shown that overexpression of PKCε protects MCF-7 cells against TNF-induced apoptosis [7,8,21]. Since PKCε also regulates autophagy, we examined the impact of PKCε-mediated autophagy on apoptosis. TNF not only caused a concentration-dependent increase in poly (ADP-ribose) polymerase (PARP) cleavage, but also an increase in LC3-II (Figure 8). While overexpression of PKCε attenuated PARP cleavage as judged by the ratio of cleaved PARP versus total PARP, it enhanced LC3-II levels. TNF also enhanced cleavage of p62, which is a substrate for caspase-8 and -6 [27,28]. We then examined if inhibition of autophagy potentiates apoptosis. Knockdown of Atg5 decreased LC3-II with a concomitant increase in LC3-I. Atg5 knockdown also enhanced TNF-induced cleavage of PARP and p62 in both MCF-7/Neo and MCF-7/PKCε cells. These results suggest that overexpression of PKCε protects cells against apoptosis by inducing autophagy.

## 3. Discussion

Protein kinase C-ε has been implicated in almost every step in the development and progression of breast cancer, including cell proliferation, epithelial-to-mesenchymal transition, invasion, and metastasis [5,29]. It is also well established that PKCε promotes breast cancer cell survival by inhibiting apoptosis [3,10]. Even though autophagy plays an important role in the development and progression of breast cancer [22], the contribution of autophagy in PKCε-mediated survival of breast cancer cells is not known. The results of our present study show that PKCε positively regulates autophagy, especially when challenged with cellular stress, such as starvation or mTOR inhibition. Moreover, PKCε-mediated induction of autophagy protects against cell death by apoptosis. 

We used isogenic cell lines and manipulated PKCε by ectopic expression or siRNA silencing to investigate the role of PKCε in autophagy. We first investigated if PKCε regulates basal autophagy, which serves as a quality control mechanism to remove damaged macromolecules and organelles [15]. Many cancer cells often upregulate autophagy to cope with increased protein turnover during cell proliferation and to survive a stressful and hostile tumor microenvironment [15]. We found that knockdown of PKCε in both T47D and MCF-7 breast cancer cells decreased autophagy as determined by the decrease in LC3-II and increase in p62. Autophagy is a dynamic process. Following autophagosome formation, autophagosomes fuse with lysosomes, and a high turnover of LC-II by lysosomal degradation can also result in a decrease in LC3-II [23,24]. We found that even though inhibition of autophagosome–lysosome fusion by Baf A1 increased LC3-II, PKCε knockdown was able to suppress this increase. These results are consistent with the report by Toton et al., where it was shown that PKCε knockdown decreased autophagy in glioblastoma cells that contained high levels of PKCε [12]. 

Our results suggest that PKCε not only regulates basal autophagy but also starvation-induced autophagy. Based on both Western blot and fluorescence microscopy, knockdown of PKCε decreased starvation-induced autophagy. While the effect of PKCε knockdown on autophagy suggests that PKCε positively regulates autophagy, the effect of PKCε overexpression on autophagy was more complex. Although PKCε overexpression caused a modest increase in LC3-II in MCF-7 cells, it also caused a substantial increase in LC3-I and an increase rather than a decrease in p62. However, when PKCε-overexpressing MCF-7 cells were starved in EBSS, the LC3-I level was decreased and LC3-II level increased in the presence of bafilomycin A1 compared to vector-transfected cells. Thus, PKCε overexpression positively regulates starvation-induced autophagy.

Since mTORC1 senses the nutrient status of cells and activates autophagy during nutrient-poor or stressful conditions, we examined if PKCε regulates autophagy via mTORC1. When mTORC1 is active, it associates with the ULK1 complex and phosphorylates ULK1 at Ser757 to inhibit autophagy [25,26]. When mTORC1 is inactive, it dissociates from ULK1, which then becomes activated by autophosphorylation to trigger autophagy [14]. We found that overexpression of PKCε increased S757-ULK1 phosphorylation, suggesting that PKCε activates rather than inhibits mTORC1. Since PKCε overexpression caused activation of mTORC1, the increase in LC3-I and p62 in PKCε-overexpressing MCF-7 cells may be a consequence of mTORC1 activation. This is consistent with our observation that the mTORC1 inhibitor rapamycin abrogated the increase in LC3-I and p62 in PKCε-overexpressing MCF-7 cells. 

mTORC1 acts downstream of the PI3K/Akt pathway, which is frequently deregulated in breast cancer [20,30]. We have previously shown that PKCε acts upstream of Akt in breast cancer MCF-7 cells to promote cell survival [7]. Since mTORC1 acts downstream of Akt, PKCε can cause activation of mTORC1 via Akt. This may explain why overexpression of PKCε increased phosphorylation of Ser757-ULK1. It is conceivable that activation of PKCε, Akt, and mTOR signaling contributes to breast cancer when cells are not dependent on autophagy to survive, but PKCε can promote cell survival by inducing autophagy when cells are challenged with stress. 

Although mTORC1 has been intimately associated with autophagy, recent studies suggest that mTORC2 is also involved in autophagy [31,32]. While mTORC1 acts downstream of Akt, both Akt and PKCε are substrates for mTORC2 [33,34]. We found that knockdown of not only Raptor but also Rictor increased LC3-II. In fact, Rictor knockdown was more effective in increasing LC3-II compared to Raptor knockdown, and depletion of PKCε counteracted the increase in LC3-II caused by Raptor or Rictor knockdown. There is a reciprocal relationship between mTORC1 and mTORC2 [30]. For example, activation of mTORC1 was shown to cause suppression of mTORC2 activity [30,35]. Thus, one possibility is that the activation of mTORC1 by PKCε results in inhibition of mTORC2, resulting in autophagy induction. However, we have previously shown that overexpression of PKCε caused an increase in Akt phosphorylation [7]. mTORC2 has several other substrates besides Akt [17] and autophagy is regulated by several kinases [36]. Both Raptor and Rictor may influence autophagy via AMPK (5’ AMP-activated protein kinase), another major regulator of autophagy [37,38]. Future studies should discern if PKCε mediates autophagy via mTORC2, AMPK, or some other kinases.

Most chemotherapeutic drugs kill cancer cells by inducing apoptosis, but the development of resistance to such therapy is a significant problem. Because the PI3K/Akt/mTOR pathway is frequently deregulated in breast cancers, mTORC1 inhibitors have been used in the clinic, but the success of rapalogues has been thwarted due to the activation of Akt involving a negative feedback loop [17]. Since PKCε is well known for its ability to inhibit apoptosis, we also examined the impact of PKCε-mediated autophagy on apoptotic signaling. We have previously shown that overexpression of PKCε in MCF-7 cells protects against TNF-induced apoptosis [7,8,21]. Our present study shows that TNF not only induced apoptosis but also induced autophagy, which was increased substantially in PKCε-overexpressing cells. Inhibition of autophagy by knockdown of Atg5 restored the ability of MCF-7/PKCε cells to induce apoptosis. Since overexpression of PKCε contributes to chemoresistance, targeting autophagy in combination with standard-of-care treatments may be effective in treating breast cancers in which PKCε is overexpressed.

## 4. Materials and Methods 

### 4.1. Reagents

Polyclonal antibody against LC3B and phosphorylated antibodies were purchased from Cell Signaling Technology (Danvers, MA). Polyclonal antibody against PKCε and monoclonal antibody against p62 were obtained from Santa Cruz Biotechnology, Inc. (Santa Cruz, CA, USA). Monoclonal antibody against PARP was obtained from BD Pharmingen (San Diego, CA, USA). Monoclonal antibodies against actin and tubulin, and chloroquine diphosphate salt were purchased from Sigma (St. Louis, MO, USA). Bafilomycin A1 and rapamycin were obtained from LC Laboratories (Woburn, MA, USA). Human recombinant TNFα was purchased from R&D Systems, Inc. (Minneapolis, MN, USA). Horseradish-peroxidase-conjugated donkey anti-rabbit and goat anti-mouse secondary antibodies were purchased from Jackson ImmunoResearch Laboratories, Inc. (West Grove, PA, USA). Polyvinylidene difluoride transfer membrane was obtained from Thermo Fisher Scientific (Waltham, MA, USA) and the enhanced chemiluminescence detection kit was from Perkin-Elmer (Shelton, CT, USA). Protease inhibitor and phosphatase inhibitor cocktails were purchased from Calbiochem/EMD-Millipore (Bedford, MA, USA). Control non-targeting and target-specific siGENOME SMARTpool siRNAs were obtained from Dharmacon (Lafayette, CO, USA). Lipofectamine RNAiMax transfection reagent was obtained from Invitrogen (Carlsbad, CA, USA).

### 4.2. Cell Culture and Transfection

T47D and MCF-7 cells were cultured in RPMI 1640 medium supplemented with 7.5% fetal bovine serum and 2 mM glutamine and kept in a humidified incubator at 37 °C with 95% air and 5% CO_2_. T47D cells stably expressing mCherry-GFP-LC3 were generated by infecting T47D cells with retroviral particles using pBABE-mcherry-GFP-LC3 vector (Addgene, Cambridge, MA, USA) and selected using puromycin. siRNA transfections were performed with 10 nM control non-targeting or target-specific siRNAs using Lipofectamine^®^ RNAiMAX transfection reagent (Invitrogen, Carlsbad, CA, USA) according to the manufacturer’s protocol. Then, 48 h following siRNA transfection, cells were treated as indicated in the text and processed for Western blot analysis. MCF-7/Neo and MCF-7/PKCε were generated as described previously [21].

### 4.3. Western Blot Analysis

Cells were lysed in extraction buffer containing 20 mM Tris-HCl, pH 7.4, 0.15 M NaCl, 1 mM EGTA, 1 mM EDTA, 1.0% Nonidet-40, 10 mM β-glycerophosphate, protease inhibitor cocktail, and phosphatase inhibitor cocktail. Equivalent amounts of total proteins (10–25 μg) were electrophoresed by SDS-PAGE and transferred electrophoretically to polyvinylidene difluoride membrane. The blots were visualized using the enhanced chemiluminescence detection reagents and the manufacturer’s protocol. The blots were probed with actin or tubulin to control for equal loading.

### 4.4. Fluorescence Microscopy

Cells were plated on 8-well chamber slides (MatTek Life Sciences, Ashland, MA, USA), transfected with or without siRNAs and treated with EBSS, Baf A1, or chloroquine as described in the text. At the end of the incubation, cells were mounted using Prolong anti-fade and visualized using a confocal microscope.

### 4.5. Statistical Analyses

The intensities of immunoreactive proteins were quantified using ImageJ software (National Institutes of Health; Bethesda; Maryland; USA). Statistical significance was determined by student’s paired *t*-test using GraphPad Prism software. A *p*-value of <0.05 was considered statistically significant.

## Abbreviations

Atg	Autophagy-related
Baf A1	Bafilomycin A1
EBSS	Earle’s balanced salt solution
MAP1LC3/LC3	Microtubule-associated protein 1 light chain 3
mTOR	Mechanistic target of rapamycin
PARP	Poly (ADP-ribose) polymerase
PKC	Protein kinase C
Raptor	Regulatory-associated protein of mTOR
Rictor	Rapamycin-insensitive companion of mTOR
SDS-PAGE	Sodium dodecyl sulfate-polyacrylamide gel electrophoresis
TNF	Tumor necrosis factor-α
ULK1	Unc-51-like kinase-1
